# Reducing thoracic and lumbar radiographs in an urban emergency department through a clinical champion led quality improvement intervention

**DOI:** 10.1186/s12873-022-00611-x

**Published:** 2022-04-29

**Authors:** Joshua Sapadin, Linelle Campbell, Komal Bajaj, Joshua B. Moskovitz

**Affiliations:** 1 Albert Einstein College of Medicine, 1300 Morris Park Ave, Bronx, NY USA; 2 Department of Emergency Medicine, Jacobi Medical Center, Albert Einstein College of Medicine, 1400 Pelham Parkway South, Bronx, NY 10461 USA; 3 Department of Obstetrics and Gynecology, Jacobi Medical Center, Albert Einstein College of Medicine, 1400 Pelham Parkway South, Bronx, NY 10461 USA; 4 Department of Public Health, Hofstra University School of Health Sciences, Hempstead NY 11549 College of Medicine, 1400 Pelham Parkway South, Bronx, NY 10461 USA

**Keywords:** Low back pain, Emergency department, Radiographs, X-rays, Thoracic, Lumbar, Appropriate, Choosing wisely, Medical overuse, Quality improvement

## Abstract

**Background:**

Low back pain is a common emergency department (ED) complaint that does not always necessitate imaging. Unnecessary imaging drives medical overuse with potential to harm patients. Quality improvement (QI) interventions have shown to be an effective solution. The purpose of this QI intervention was to increase the percentage of appropriately ordered radiographs for low back pain while reducing the absolute number.

**Methods:**

A multi-component intervention led by a clinician champion including staff education, patient education, electronic medical record modification, audit and peer-feedback, and clinical decision support tools was implemented at an urban public hospital Emergency Department. In addition to the total number ordered, Choosing Wisely and American College of Radiology recommendations were used to assess appropriateness of all ED thoracic and lumbar conventional radiographs by chart review over eight months.

**Results:**

The percent of appropriately ordered radiographs increased from 5.8 to 53.9% and the monthly number of radiographs ordered decreased from 86 to 47 over the eight-month initiative. There were no compensatory increases in thoracic or lumbar computed tomography (CT) scans during this time frame.

**Conclusion:**

A multi-component QI intervention led by a clinician champion is an effective way to reduce the overutilization of thoracic and lumbar radiographs in an urban public hospital emergency department.

## Background

Low back pain is one of the most common presenting complaints in U.S. Emergency Departments (ED) [1]. Attempts to quantify the prevalence of low back pain suggest there are between two and three million ED visits annually, accounting for almost 4.4% of all ED visits in the U.S. [2, 3]. In addition to a history and physical, evaluation of low back pain in the ED often includes imaging tests. However, overuse of medical imaging for back pain is widely recognized as a problem and a growing concern [4].

There is potential for patients harm from overutilization through additional testing and imaging, incidental findings, increased length of stay, unnecessary follow-up referrals, invasive procedures, exposure to excess radiation, increased likelihood of surgical interventions, and increased patient and institutional cost [5]. Studies of care for low back pain correlate imaging as an important driver of non-financial costs and have shown this image overutilization fails to improve clinical outcomes [6]. The average cost of an ED visit in the U.S. ranges from $290–690 [7], but if the chief complaint is back pain and requires imaging the cost can be as high as $2000 [8]. The cost of thoracic and lumbar radiographs vary widely across the country in the range of $100 – $1000 [9]. Whether paid by patient, insurance company, or facility itself; inappropriate studies confer little to no benefit to the patient. Unnecessary imaging studies are therefore a conduit for the delivery of low-value care.

The decision to order imaging tests when a patient presents with low back pain depends on a detailed history and physical examination as well as the presence of “red flags”, signs and symptoms identifying high risk conditions such as trauma, cancer, infection, or spinal cord involvement. In the absence of “red flag” indicators, non-traumatic acute low back pain less than four weeks in duration generally self resolves and does not necessitate imaging nor intervention [10].

Medical overuse is a complex problem, with some clear common drivers. Physician cited motivators are fear of missing a diagnosis, fear of litigation, and perceived improved patient satisfaction. The patient motivators most overwhelmingly cited is their desire for imaging, irrespective of their knowledge of the risks of ionizing-radiation [11]. Evidence suggests multi-component interventions are successful at implementing clinical guidelines that reduce the use of a service. Examples of successes include decreased utilization of antibiotics [12], radiographs in the ICU [13], and cardiac imaging [14].

We hypothesized that a multi-component intervention led by a clinician champion in the ED over the course of eight months would increase the proportion of thoracic and lumbar radiographs ordered that meet appropriateness criteria and decrease the absolute number of those imaging tests ordered.

## Methods

This was a prospective quality improvement (QI) interventional study of all patients receiving conventional lumbar and thoracic radiographic imaging in our Emergency Department from June 2019 to January 2020. The primary outcome was the percentage of appropriately ordered radiographs. The secondary outcome was the absolute number of radiographs completed.

### Ethics and approval to consent

The study was performed in accordance with the Declaration of Helsinki and methods were performed in accordance with the relevant guidelines and regulations. The study was approved by the Albert Einstein College of Medicine Institutional Review Board #2020–11,068 under the waiver of informed consent and HIPAA authorization. This manuscript was prepared in accordance with the Standards for Quality Improvement Reporting Excellence (SQUIRE 2.0) [15] guidelines for the reporting of quality improvement work.

### Study setting and population

#### Setting

Safety net urban hospital Emergency Department in the Bronx, New York, with a large Emergency Medicine Residency Program having >100,000 annual visits. Staffing in our lower acuity zone (two residents, two advanced practice providers (APPs), and two attendings from 7 am-11 pm) where most non-traumatic low back pain patients are evaluated, and where our intervention was more concentrated. The hospital is a level one trauma center serving a limited resource population and a member of the nation’s largest public health hospital system. More than 70% of these patients identify as either Hispanic or African American, and almost a third are uninsured on arrival. In our adult emergency department, “back pain” constitutes approximately 5% of all chief complaints and over 2% of our discharge diagnoses.

#### Study population

Patients 21 years and older presenting to the ED from June 2019 to January 2020 whom received a thoracic or lumbar conventional radiograph were identified by radiology work list review and chart review. There were no exclusion criteria.

#### Intervention

Our intervention had five components: physician education, patient education, a clinical decision support tool, audit and feedback, and Electronic Medical Record (EMR) modification. The entire intervention for this QI initiative was led by one of the authors who served as the clinician champion in the ED.

**Fig. 1:**
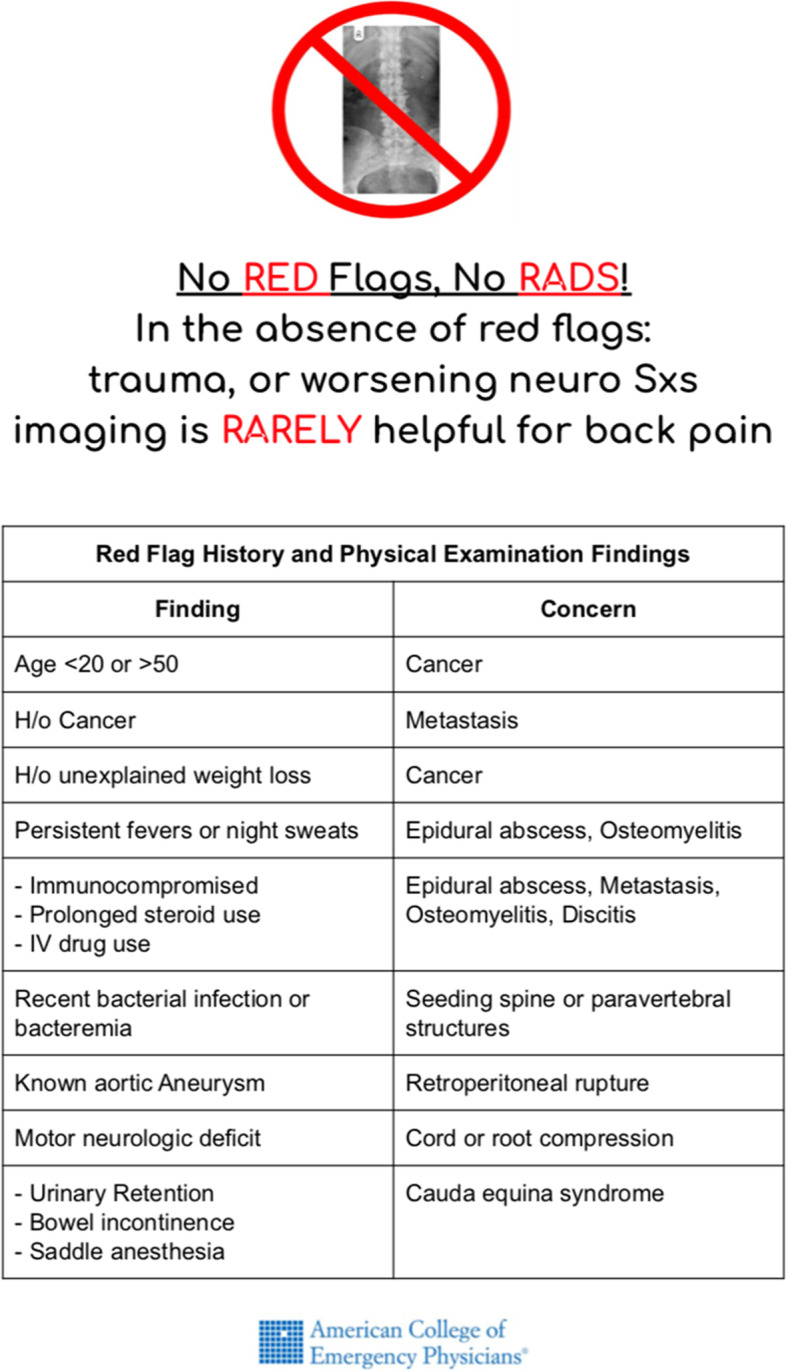
Best Practice Advisory card containing red flag indicators for imaging of low back pain

1. *Physician and provider education*
  Education of the physicians and advance practice providers were done in several stages over time. At resident educational conferences two separate sessions were conducted. “What is medical overuse and why does it create harm?” was presented in May 2019 (one month prior to the start of the intervention) to garner interest in the topic and recruit resident clinician champions, and “Evaluating Low Back Pain in the Urban Emergency Department” in August 2019 (two months into the intervention). The topic was also discussed and reviewed at bi-weekly faculty meetings.
2. *Patient education*
  Patient education was implemented in three phases beginning at the start of the intervention (June 2019). First, when registering for care, patient education material in both English and Spanish about low back pain evaluation from the Choosing Wisely website was available for patients to pick up on their own [16]. Second, the same educational material was posted on the walls of patient rooms in the low acuity zone of the ED. Third, face-to-face education of the patients was done by the treating physician. In all phases, patient education consisted of presenting the indications for radiographs, the risks of imaging without red flag indicators as well as expected disease courses.
3. *Clinical decision support tool*
  We used the Choosing Wisely (CW) [17] and the American College of Radiology (ACR) [18] recommendations for low back pain imaging to develop “Red Flag” indicators for low back pain imaging in the setting of low back pain in the ED. All providers were given a clinical decision support tool in the form of a best practice advisory (BPA) card (Fig. 1) with these Red Flag indicators in their mailboxes and at faculty and at residency conferences. The BPA cards are approximately two by three-inch laminated cards that can easily fit into a lab coat or scrub pocket. In addition, BPA cards were physically attached to the upper right-hand corner of each computer in the ED where providers would be entering orders. The intent of BPA cards was to serve as an easily accessible clinical reminder when determining the need to order imaging for low back pain patients. The BPA cards were redistributed every other month as needed.

4. *Audit and feedback*
  At the beginning of each month, the highest utilizers of radiographic imaging for low back pain from the prior month were identified via audit of the EMR: five attending physicians and five residents or Advanced Practice Providers. Feedback was given to each provider in the form of a face-to-face discussion involving a review of the BPA card material. The feedback was conducted by one of the authors based on a prior existing relationship with the highest utilizers.

5. *EMR Modification*
  Within the EMR of our ED, we have departmental favorite orders that are available on a quick list intended to save time when placing common orders. We removed the quick-order option for thoracic and lumbar radiographs, thereby necessitating providers to search for the order manually if needed.

### Data collection and analysis

Data on thoracic or lumber radiographs for patients with low back pain was obtained from the Department of Radiology logs of imaging performed in the ED from three months prior to beginning the QI initiative until eight months after. In addition to radiographs, counts of thoracic or lumbar CT scans completed on patients with low back pain seen in the ED were collected during the same time interval. This was done to assess if providers were replacing radiograph orders with CT orders. Magnetic Resonance Imaging is not readily available in our ED, reserved only for emergencies, and typically necessitates an inpatient hospitalization and therefore was not assessed for this project.

Medical record numbers from the logs were used to identify patients in the EMR for evaluation of imaging appropriateness. Using CW [7] and ACR [8] criteria for imaging in the setting of back pain, we defined appropriateness as back pain lasting less than four weeks with at least one red flag (Fig. 1). To determine appropriateness a chart review was done on all patients who received conventional lumbar and thoracic radiographs in our ED using these appropriateness criteria. Chart review was split evenly and conducted by three residents and one attending physician. Each chart reviewer had 10% of their charts reviewed by a second reviewer to assess inter-rater reliability, of which 100% was noted.

The proportion of patients with an appropriate thoracic or lumbar radiograph was calculated and reported by month, along with a total count of the number of low back pain patients with a thoracic or lumbar radiographs or CT scan ordered. As a QI initiative, no statistical tests of significance were performed because of the lack of a control group.

## Results

**Fig. 2:**
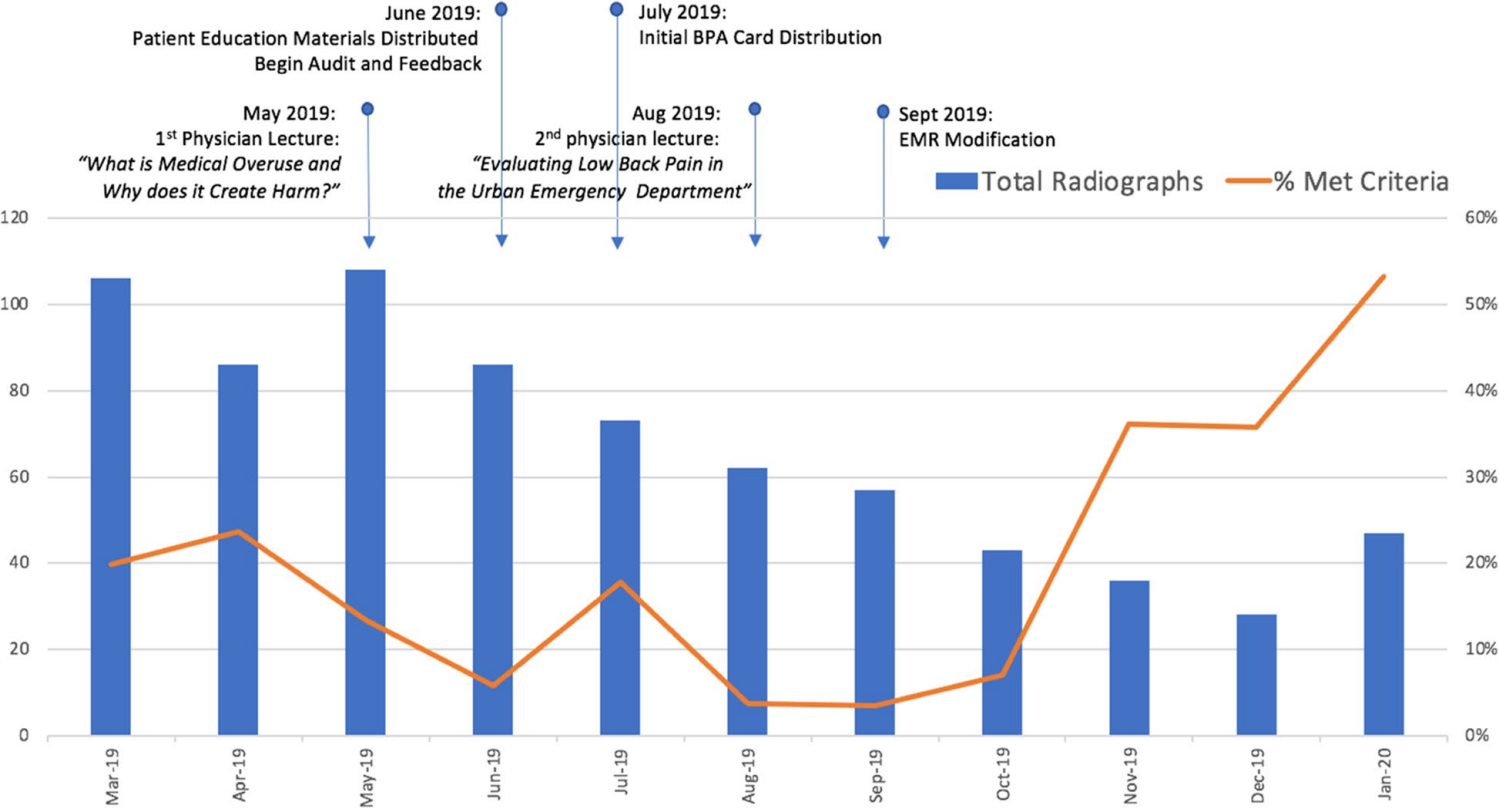
Monthly radiographs and percent that met appropriateness criteria

**Fig. 3:**
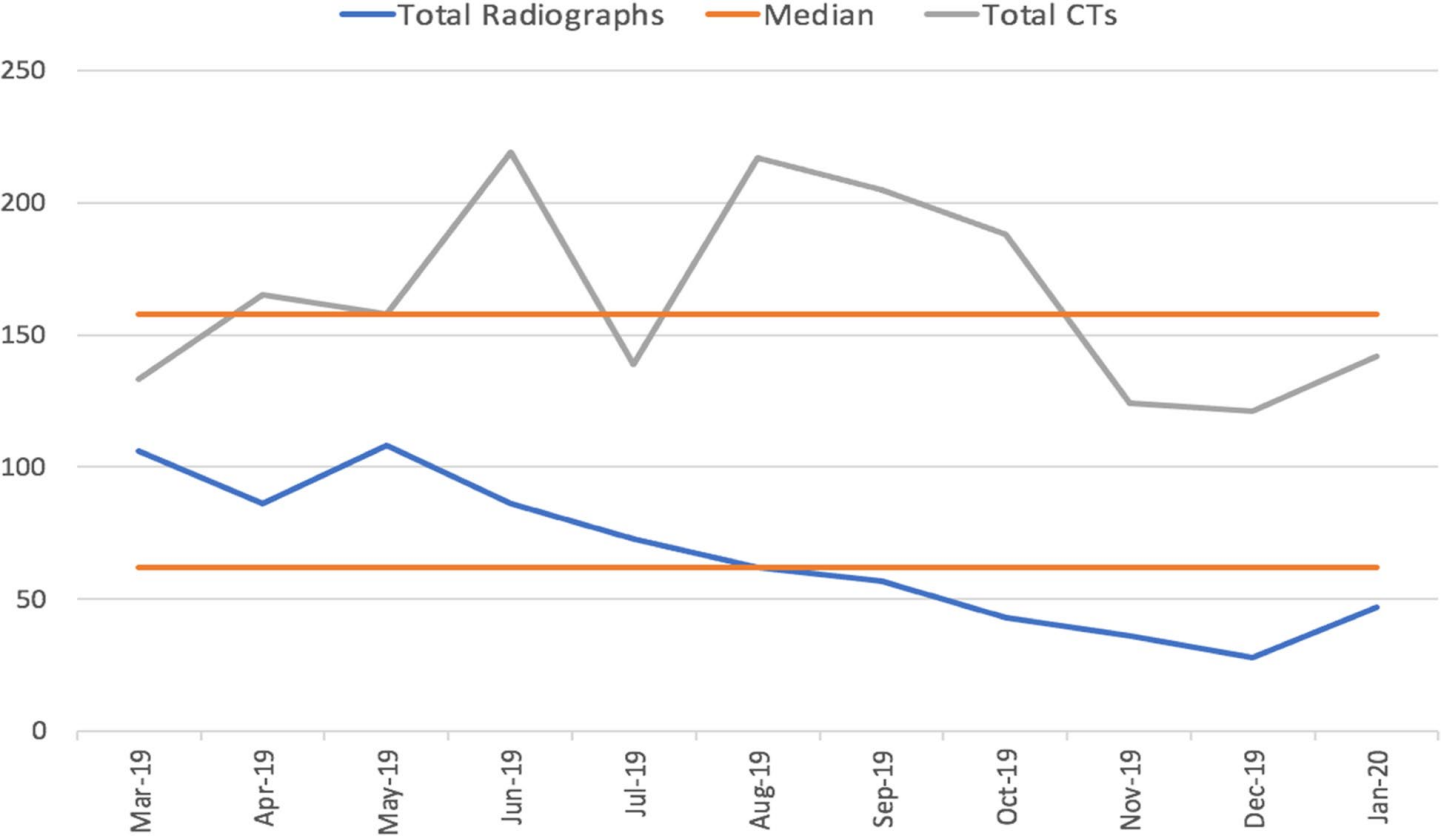
Absolute monthly radiographs and CT scans

Over the course of the eight-month intervention a total of 432 thoracic and lumbar radiographs were performed and the absolute number of radiographs ordered monthly decreased from 86 to 47 (Fig. 2). This compares to an average monthly number of 90 thoracic or lumbar radiographs ordered over the three months prior to the QI initiative. The percent of appropriate radiographs ordered increased from 5.8 to 53.2% over the same period. A separate count of the number of computed tomography (CTs) of the thoracic and lumbar spine performed for low back pain demonstrated no evidence of replacing radiographs with CT scans (Fig. 3).

## Discussion

A multi-component QI intervention led by a clinician champion resulted in a reduction in the number of radiographs ordered to evaluate low-back pain and an increase in the proportion of those ordered that were appropriate. In addition, there was no evidence that these imaging tests were replaced by available alternative imaging. To our knowledge this is the first evaluation of a clinician champion lead initiative that addresses thoracic and lumbar radiograph reduction in the ED setting using a multi-component QI intervention. By engaging clinicians through education, audit and feedback and providing them with a clinical decision support tool, the total number of unnecessary radiographs ordered decreased by almost 44% and of those that were ordered, the proportion that were appropriate increased by 10-fold. Over eight months we demonstrated through a multi-component intervention that this could be addressed and substantially reduced.

The results demonstrate some fluctuation in the appropriateness criteria on its trajectory of overall improvement and there may be a few causes. Our health system transitioned our hospital to a new electronic medical record the very end of July which could have made documentation more challenging the first month afterwards. Our hospital has a very significant presence of resident physicians, who rotate through the emergency department monthly necessitating repetitive in-servicing of quality initiatives. We also believe the quality of documentation improved overall from this initiative thus not having a true reverse linear relationship between appropriateness and absolute reduction.

Our results are consistent with previous studies which highlight evidence that QI interventions can address the issue of reducing image utilization. A QI intervention at a high-volume pediatric ED in California used similar methods to reduce abdominal radiographs for patients presenting with constipation [19]. Relying heavily on physician education in the form of lectures, and audit and feedback, they reduced the number of pediatric patients that received unnecessary abdominal radiographs by 50% over a 12-month period. Another QI intervention in a pediatric ED sought to reduce chest radiographs for patients presenting with bronchiolitis [20]. They employed physician education lectures and posted imaging guidelines at each computer station, and reduced radiographs for bronchiolitis patients by 44% at the end of a four-month period.

The dissemination of official guidelines alone may lead to successful outcomes in QI interventions. However, it has been shown that the addition of clinician champions led to higher rates of success. A literature review studying interventions that attempted to reduce low value care defined by Choosing Wisely found that interventions led by clinician champions reached their intended outcomes 71% of the time [21]. This underscores the importance of clinician-led initiatives when attempting to improve quality of care.

Concerned the reduction of radiographs could have the unintended consequence of an increase in CT scans of the thoracic and lumbar spine we assessed the absolute number ordered as well. As a level one trauma center that does a significant number of these CT scans, there were no demonstrated special cause variation around the median during our QI project. Further, the number of radiographs in the three months prior to the intervention demonstrate stable common cause variation.

The average physician to disposition time for patients presenting with back pain being discharged from our emergency department is approximately ninety minutes. A conventional radiograph from ordered to read time also averages approximately ninety minutes. This implies that by decreasing the utilization of radiographs, the patient throughput time improves dramatically which also has an outlying effect on the door to first provider time by freeing up space in the ED for patient evaluation and treatment.

### Limitations

A limitation of this intervention is the lack of a control group. Without a control, we cannot be certain that this was not just a natural phenomenon that would have occurred without the intervention. ED volumes are dynamic and vary monthly due to multiple factors, and our study period was limited from June to January in an academic emergency department, making it possible that other factors could affect the number of radiographs ordered.

Low rates of radiograph appropriateness prior to the intervention could be due to poor documentation, but there was demonstrated consistent improvement at the same time of absolute reduction. Our institution changed from a legacy EMR (Quadramed) which utilized blank word type documents to EPIC the last week of July which may have had an impact on documentation thoroughness. It was not possible to calculate the rates of x-ray utilization in our diverse level one trauma emergency department automatically and would necessitate a substantial manual chart abstraction thus it was chosen to evaluate x-rays performed for appropriateness.

It is also possible that the observed impact of our intervention could be explained by the Hawthorn effect, in which one’s awareness they are being audited leads to changes in their behavior to improve their performance.

We did not measure the prolonged effects of this initiative to measure full sustainment but have used this opportunity to parlay additional clinician champion initiatives in other modalities leading to sustained awareness of overuse as a medical concern.

## Conclusion

We conclude a multi-component intervention led by a clinician champion is an effective means to reduce utilization of thoracic and lumbar conventional radiographic imaging in a public hospital ED for patients with non-traumatic acute low back pain. Further research is needed to validate these findings and explore the sustainability of these results.

Additional research quantifying unnecessary imaging to subsequent incidental findings, procedures, follow-up, and lengths of stay in the hospital would further the value of using QI interventions to improve the delivery of high-quality care.

Healthcare expenditures are continually on the rise, and with an aging population this will continue to increase. Reducing medical overuse in the ED is an opportunity to reduce the burden of disease on patients, facilities, and systems. While ED visits are increasing, ED imaging is increasing disproportionately faster [22]. This leads to opportunities for intervention and improved patient outcomes.

## Data Availability

The datasets used and/or analyzed during the current study are available from the corresponding author on reasonable request.

## Abbreviations

ACR: American College of Radiology
APP: Advanced Practice Providers
BPA: Best Practice Advisory
CT: Computed Tomography
CW: Choosing Wisely
ED: Emergency Department
EMR: Electronic Medical Record
QI: Quality Improvement

## References

[1] National Center for Health Statistics. National Ambulatory Medical Care Survey: 2017 State and National Summary Tables. Centers Dis Control Prev. 2017. 10.1080/0308107031000075690.

[2] Friedman BW, Chilstrom M, Bijur PE, Gallagher EJ (2010). Diagnostic testing and treatment of low back pain in United States emergency departments: a national perspective. Spine (Phila Pa 1976).

[3] Edwards J, Hayden J, Asbridge M, Gregoire B, Magee K. Prevalence of low back pain in emergency settings: A systematic review and meta-analysis. BMC Musculoskeletal Dis. 2017;18(1). 10.1186/s12891-017-1511-7 10.1186/s12891-017-1511-7 PMC5379602 28376873

[4] Mafi JN, McCarthy EP, Davis RB, Landon BE. Worsening trends in the management and treatment of back pain. JAMA Int Med. 2013. 10.1001/jamainternmed.2013.8992. 10.1001/jamainternmed.2013.8992 PMC4381435 23896698

[5] Newton EH. Addressing overuse in emergency medicine: evidence of a role for greater patient engagement. Clin Exp Emerg Med. 2017. 10.15441/ceem.17.233. 10.15441/ceem.17.233 PMC5758625 29306268

[6] Chou R, Fu R, Carrino JA, Deyo RA. Imaging strategies for low-back pain: systematic review and meta-analysis. Lancet. 2009. 10.1016/S0140-6736(09)60172-0. 10.1016/S0140-6736(09)60172-0 19200918

[7] Moore BJ. Costs of Emergency Department Visits in the United States, 2017. Costs of emergency department visits in the United States, 2017 #268. 2020; Retrieved February 1, 2022, from https://www.hcup-us.ahrq.gov/reports/statbriefs/sb268-ED-Costs-2017.jsp

[8] Jorgensen DJ (2007). Fiscal analysis of emergency admissions for chronic back pain: A pilot study from a Maine Hospital: Table 1. Pain Medicine.

[9] https://www.healthcost.com/search/72080/New%20York,%20USA/43.2994285,-74.21793260000001 Accessed 21 May 2021

[10] Chou R, Qaseem A, Snow V, Casey D, Cross TJ, Shekelle P, et al. Diagnosis and treatment of low back pain: a joint clinical practice guideline from the American College of Physicians and the American pain society. Ann Int Med. 2007. 10.7326/0003-4819-147-7-200710020-00006. 10.7326/0003-4819-147-7-200710020-00006 17909209

[11] Griffith J, Monkman H, Borycki E, Kushniruk A. Physician experiences with perceived pressure to order diagnostic imaging services. Stud Health Technol Inform. 2015. 10.3233/978-1-61499-574-6-20. 26262521

[12] Haran JP, Goulding M, Campion M, Scully G, Chandra A, Goldberg R, Day A, McLendon E, Clark MA (2020). Reduction of inappropriate antibiotic use and improved outcomes by implementation of an algorithm-based clinical guideline for nonpurulent skin and soft tissue infections. Ann Emerg Med..

[13] Scott J, Waite S, Napolitano A. Restricting daily chest radiography in the intensive care unit: Implementing evidence-based medicine to decrease utilization. J Am College Radiol. 2021;8(3):354–360. 10.1016/j.jacr.2020.05.035 10.1016/j.jacr.2020.05.035 PMC7346804 32653273

[14] Bhatia RS, Milford CE, Picard MH, Weiner RB (2013). An educational intervention reduces the rate of inappropriate echocardiograms on an inpatient medical service. JACC Cardiovasc Imaging..

[15] Davies L, Goodman D, Batalden P, Davidoff F, Stevens D (2015). Squire 2.0 (Standards for Quality Improvement Reporting Excellence): revised publication guidelines from a detailed consensus process. Am J Crit Care.

[16] Foundation ABIM. Imaging tests for lower-back pain: Choosing wisely. Choosing Wisely | Promoting conversations between providers and patients. 2018; Retrieved September 23, 2021, from https://www.choosingwisely.org/patient-resources/imaging-tests-for-lower-back-pain/

[17] https://www.choosingwisely.org/clinician-lists/acep-lumbar-spine-imaging-in-the-ed/ Accessed 28 May 2021

[18] https://acsearch.acr.org/docs/69483/Narrative/ Accessed 28 May 2021

[19] Gabriela M, et al. Reducing Abdominal Radiographs to Diagnose Constipation in the Pediatric Emergency Department. J Pediatr. 2020;225. 10.1016/j.jpeds.2020.06.028. 10.1016/j.jpeds.2020.06.028 32553869

[20] Reiter J (2018). A quality improvement intervention to reduce emergency department radiography for bronchiolitis. Respir Med.

[21] Cliff BQ, Avanceña ALV, Hirth RA, Lee SD. The impact of choosing wisely interventions on low-value medical services: a systematic review. Milbank Q. 2021. 10.1111/1468-0009.12531 Epub ahead of print. PMID: 34402553. 10.1111/1468-0009.12531 PMC8718584 34402553

[22] Iglehart JK. The new era of medical imaging - Progress and pitfalls. New Engl J Med. 2006. 10.1056/NEJMhpr061219. 10.1056/NEJMhpr061219 16807422

